# Soybean okara: a sustainable and cost-effective alternative for potato dextrose agar and nutrient agar media

**DOI:** 10.1016/j.mex.2026.103941

**Published:** 2026-05-03

**Authors:** Hemant Singh Maheshwari, Sanjeev Kumar, Laxman Singh Rajput, Aayushi Singh, Palak Solanki, Rajesh Jewaliya, Aakash Gour, Jeberlin Prabina Bright, Mahaveer Prasad Sharma, Kunwar Harendra Singh

**Affiliations:** a ICAR-National Soybean Research Institute, Indore, India; b University of Groningen, the Netherlands; c ICAR-Central Arid Zone Research Institute, Jodhpur, India; d Devi Ahilya Vishwa Vidyalaya, Indore, Madhya Pradesh, India; e Rajmata Vijayaraje Scindia Krishi Vishwavidyalaya, Gwalior, India; f Tamilnadu Agricultural University, Coimbatore, India

**Keywords:** Okara bacterial (OB) agar, Okara fungal (OF) agar, Nutrient agar (NA), Potato dextrose agar (PDA), Growth measurements, Production economics, Waste minimization, Sustainability

## Abstract

In the present method, we used okara, a by-product of the tofu or soya milk industry, as a cost-effective alternative to nutrient agar (NA) and potato dextrose agar (PDA), which are routinely used for bacterial and fungal cultivation in microbiology labs worldwide. Here, two okara-based media, namely okara bacterial (OB) and okara fungal (OF), were prepared by adding dextrose at 1 % and 2 %, respectively. Then, three distinct series of experiments were carried out to evaluate the use of okara as a microbiological media. Three different genera of bacteria and fungi were used, and growth experiments were carried out. Bacterial growth was measured through spectrophotometric absorbances, whereas fungal growth was measured based on diameter and biomass production. Further, we used okara-based media for the isolation and enumeration of bacterial and fungal populations from rhizosphere and endosphere. Finally, production economics were compared between okara-based media and readily available media.

• Okara-based media supports significantly higher fungal growth and similar bacterial growth compared to readily available media.

• Isolation and enumeration of bacteria and fungi with Okara media are possible.

• Okara-based media was >85 % cost-effective than readily available media.

• Okara-based media can be used for mass multiplication of beneficial bacteria and fungi.

## Graphical abstract

**Figure:**
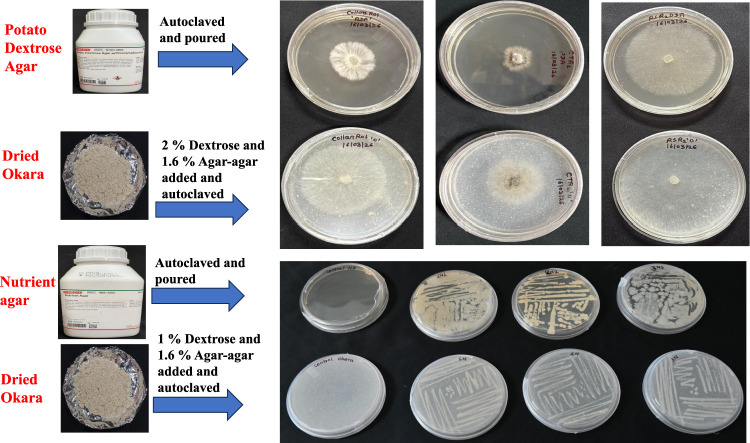

## Specifications table

**Table:** 

Subject area	Agricultural and Biological Sciences
**More specific subject area**	Agricultural Microbiology
**Name of your method**	Soybean okara: a cost-effective alternative for potato dextrose agar and nutrient agar media
**Name and reference of the original method**	NA
**Resource availability**	Okara, Agar-agar, Potato dextrose media, Nutrient agar media, Dextrose, Sodium chloride, Nystatin, and Chloramphenicol

## Background

Nutrient agar and potato dextrose agar are widely used worldwide for the growth and cultivation of a wide range of bacteria and fungi, respectively [1]. Key constituents of NA are peptone, yeast extract, beef extract, and sodium chloride. Additionally, key constituents of PDA are potato infusion, dextrose, and agar-agar. Some soybean-based media include tryptic soy agar and soybean casein digest agar media, used for the cultivation of microorganisms [2]. The average cost of these media in markets is approximately 1000 USD or more per Kg, which is expensive to use, especially for schools and colleges in developing countries.

In the past, cereal meal extracts of sorghum, millet, and corn were prepared, and different fungal genera were grown on these media and compared with PDA; fungal growth was similar or better than on PDA, except for a few genera [3]. Similarly, media prepared from cereal and legume flours, including rice, maize, chickpea, and some processed flours, have been used for cultivating bacteria and fungi, and a few genera showed greater growth in these flours [4]. Rice bran dextrose agar was prepared by adding rice bran and varying concentrations of dextrose and agar, which supported greater growth of *Aspergillus* and *Candida* sp. than PDA media [5]. However, the only limitation was that the above media were prepared through food sources shared by humans.

In the present methods, we used okara, a by-product of the tofu or soybean milk industry, which is produced annually in large quantities and poses a serious environmental concern due to its disposal into the environment [6]. One kilogram of soybeans produces approximately 1.1–1.2 kg of okara during soymilk or tofu preparation [7]. Okara contains insoluble cellulose and hemicellulose fiber (40 % - 60 %), simple carbohydrates (4 % - 5 %), 15.2 % - 33.4 % protein, 8.3 % - 10.9 % lipids, and many other nutrients, such as starch, potassium, vitamin B, and phytochemicals [6,8]. Further, okara contains essential amino acids such as leucine, isoleucine, valine, threonine, histidine, phenylalanine, and methionine, as well as non-essential amino acids such as glutamic acid, aspartic acid, tyrosine, and serine [9]. Currently, it is used for the preparation of medicines due to its prebiotic potential, in the food industry, and as a fermentation substrate [6]. Further, it has been used in the preparation of some dishes and is recommended for adding to wheat flour to supplement protein in the human diet.

Microbial cultivation in the laboratory requires nutrients comprising various carbon and nitrogen sources, organic and inorganic nutrients, trace elements, and growth factors [2]. To the best of our knowledge, we did not find any studies using okara in the preparation of microbiological media that support the growth and cultivation of bacteria and fungi. We hypothesized that okara is rich in all nutrients except simple sugars; therefore, the addition of dextrose and the solidifying agent agar-agar to dried okara may support both bacteria and fungi. Therefore, we prepared two different media, namely OB and OF, for the growth of bacteria and fungi, respectively, using dried okara and supplementing the constituents lacking in it; bacterial and fungal growth were then measured, and these two media were used for the isolation of bacteria and fungi from the plant rhizosphere and endosphere.

## Method details

### Equipment and consumables required

Hot air oven.

Laminar air flow.

BOD incubator shaker. pH meter.

Micropipette (100–1000 µl) single-channel capacity.

Analytical balance 220 g/0.1 mg capacity.

Erlenmeyer culture flask with flat bottom (250 mL) capacity.

Bacteriological filter 0.22 µm.

Nystatin (Himedia Laboratory, India).

Chloramphenicol (Himedia Laboratory, India).

Nutrient broth (Himedia Laboratory, India; GM1269).

Potato dextrose broth with chloramphenicol (Himedia Laboratory, India; MP5431).

Agar-Agar, Type I (Himedia Laboratory, India; GRM 666).

D-dextrose (Himedia Laboratory, India; GRM077).

Sodium chloride (Himedia Laboratory, India; TCO46).

Ethanol (70 %).

NaOH.

HCl.

Dimethyl Sulfoxide (DMSO) ACS, 99.9 % (28,580 SRL).

Cork borer.

Inoculating loops and needles.

Mortar and Pestle.

Muslin cloth.

Whatman filter paper.

Glass funnel.

Autoclavable micro pipette tips (1 mL).

Petri dishes (90 mm × 15 mm).

Cell spreaders.

Mixer Grinder.

### Antibiotics preparations

Nystatin (PCT1112, Himedia Laboratory, India) was available as a powder. A stock solution (50 mg mL^-1^) was prepared by dissolving 500 mg in 9 mL of dimethylsulfoxide (DMSO) in a 15 mL Eppendorf tube, mixing completely by shaking, and then adjusting the final volume to 10 mL with DMSO. It was filter-sterilized through a bacteriological filter membrane with a pore size of 0.22 µm, placed into another sterile 15 ml Eppendorf tube, wrapped in aluminium foil to avoid direct exposure to light, and stored in a −20 °C deep freezer. It was added to the media at a concentration of 50 µg mL^-1^ to avoid fungal contamination.

Chloramphenicol (Himedia Laboratory, India) was available in powdered form and its stock solution (30 mg mL^-1^ in 70 % ethanol) was prepared by dissolving 300 mg chloramphenicol in 9 ml of 70 % ethanol into 15 ml Eppendorf tube and mixed until dissolve completely and volume adjusted to 10 ml finally with 70 % ethanol and then filter-sterilized through a bacteriological filter (0.22 µm) and placed into another sterile 15 ml Eppendorf tube, wrapped in aluminium foil to prevent exposure to light, and stored at −20 °C deep freezer. Chloramphenicol was added to the okara fungal media at 170 µg mL^-1^ to avoid bacterial contamination.

### Preparation of okara-based media

Soybean okara was collected as a byproduct of the tofu-making process, prepared from yellow soybeans, dried in an oven at 65 °C for 24 h, and then ground to a powder consistency using mixer grinder at ICAR-National Soybean Research Institute, India. Okara fungal (OF) and okara bacterial (OB) media were prepared using the following constituents and procedures:

• **OF media:** OF media composition (g l^-1^) was prepared using the following: okara powder-10 g l^-1^, dextrose-20.00 g l^-1^, and agar-agar-16.00 g l^-1^. Here, 10 g of dry powdered okara was mixed with 950 mL of double-distilled water in a beaker, then placed in a microwave at 100 °C for 2 min to ensure complete dissolution. Then it was strained through a muslin cloth to remove any undissolved soybean fiber or black hilum. Finally, dextrose was added, and pH was maintained at 5.60 at 25 °C. Distilled water was added, and the final volume was made to 1000 mL for the okara fungal broth media.

For the okara fungal agar medium, agar-agar was added after adjusting the pH, and the final volume was adjusted to 1000 ml with distilled water. Then, the media was autoclaved at 121 °C for 20 min and cooled to approximately 42–44 °C at room temperature. Then, filter-sterilized chloramphenicol was added to the media at 170 µg mL^-1^ from the stock solution.

The dextrose concentration in OF medium was kept at 2 %, similar to that of potato dextrose agar media.

• **OB media:** OB media was prepared using the following: Okara- 10 g l^-1^, NaCl- 5.00 g l^-1^, dextrose- 10.00 g l^-1^, and agar-agar- 16.00 g l^-1^. Here, 10 g of okara was added to 950 ml of double-distilled water, heated in a microwave at 100 °C for 2 min to ensure the powder was fully dissolved, and then strained through muslin cloth to remove any undissolved okara. Then, dextrose was added, the pH was maintained at 7.60 at 25 °C, and the final volume was adjusted to 1000 ml with distilled water for the liquid medium. For a solid medium, agar-agar was added after adjusting the pH, and the final volume was adjusted to 1000 ml with distilled water. It was autoclaved at 121 °C for 20 min, cooled to approximately 42–44 °C at room temperature, and then nystatin at 50 µg mL^-1^ was added from a stock solution.

Since the readily made media, namely Tryptone glucose yeast extract broth (M952, Himedia Laboratory) and Glucose yeast extract BC agar medium (M2102, Himedia Laboratory), contained 0.5 % glucose. Similarly, Glucose peptone agar (M758, Himedia Laboratory) and Dextrose agar (M084, Himedia Laboratory) contained 1 % glucose. Therefore, in OB medium, we tested at two different glucose concentrations (0.5 % and 1 %). However, bacterial growth at 0.5 % glucose in Okara medium was slower than in NA; therefore, we used 1 % glucose for bacterial growth experiments.

1) **Bacterial and fungal growth measurements**
  a) **Fungal growth measurements**
    • Three different fungi with five-day-old cultures, namely fast grower *Rhizoctonia solani* and *Sclerotium rolfsii*, and slow grower *Colletotrichum truncatum*, were taken from ICAR-NSRI, Plant Pathology Lab.
    • **Inoculum preparation:** These cultures were freshly grown by inoculating the respective fungal cultures into potato dextrose agar medium and incubating at 28 ± 2 °C. These 5-day-old, freshly grown cultures were then used for further fungal growth experiments to ensure uniform physiological characteristics.
    • **Fungal diameter measurements:** Using a cork borer, 10 mm diameter fungal discs were aseptically cut and centrally inoculated onto Petri plates containing OF agar, with PDA used as the control. It was kept in the BOD incubator at 28 ± 2 °C. Then, the fungal diameters were recorded when the fungi completely filled the Petri dish [3]. The study was carried out using a completely randomized design (CRD) with 8 replications.
    • **Fungal biomass measurements**: Here, 250 mL conical flasks containing 100 mL OF broth were prepared and autoclaved. Using a cork borer, a 10 mm fungal phytopathogen disc was inoculated aseptically. Similarly, the same setup was carried out with potato dextrose broth (PDB) media as a control. The conical flasks were incubated in a BOD incubator shaker at 28 ± 2 °C for 5 days with continuous agitation at 80 rpm. The study was carried out using a CRD with 8 replications. Fungal dry biomass was measured after decanting OF broth and PDB, and the mycelium was then oven-dried at 80 °C for 48 h to obtain the dry weight [10].
  b) **Bacterial growth measurements**
    • Three different genera of bacteria, namely *Bacillus amyloliquefaciens, Achromobacter denitrificans*, and *Pseudomonas* sp., were taken as test bacteria from ICAR-NSRI, Microbiology Lab.
    • **Inoculum preparation:** The above bacterial cultures were initially grown in nutrient broth at 28 °C for 24 h, and the spectrophotometric absorbance was recorded at 600 nm; the optical density of bacterial suspension was set to 1.00 with sterile uninoculated NB.
    • **Colony characteristics observation**: One loop of bacteria was streaked onto OB agar and NA as controls in 8 replicates. These Petri dishes were placed into the BOD at 28 °C for 24 h. Here, we used two dextrose concentrations (0.5 % and 1 %) to prepare okara agar plates. It was performed to visually observe whether bacteria were growing on this OB agar medium.
    • **Bacterial growth measurement:** It was measured using spectrophotometric absorbance by inoculating 10 µL of freshly grown bacterial cultures each into a 30 ml test tube carrying 10 mL OB broth containing 1 % dextrose and NB aseptically in 8 replicates and kept in a BOD incubator shaker at 28 °C with 120 rpm. Then the optical density at 600 nm was recorded at 24 h.
2) **Enumeration of bacteria and fungi from the rhizosphere and the plant endosphere.**
  • **Rhizospheric bacteria and fungi isolation and enumeration:** 1 g of soybean and wheat plant rhizospheric soil was taken and serially diluted to 10^–6^, then 100 µl of 10^–6^ aliquot was uniformly spread onto OB agar and NA for bacterial isolation. Similarly, from a 10^–4^ dilution, a 100 µL aliquot was uniformly spread onto OF agar and PDA for fungal isolation. It was carried out in eight replicates in CRD design.
  • **Endophytic bacteria and fungi isolation and enumeration:** Roots of soybean and wheat were uprooted and washed with running tap water to remove soil and adhering particles. They were surface-sterilized in 2 % sodium hypochlorite solution for 2 min, followed by washing with sterile distilled water (2 times), then placed into ethanol (70 %) for 2 min, followed by washing 10 times with sterile distilled water. These surface-sterilized roots were crushed with a mortar and pestle, and a 1 mL aliquot was then serially diluted. From the 10^–6^ dilution, 100 µL was spread onto OB agar and NA for endophytic bacterial isolation. Similarly, from 10^–4^, a 100 µl aliquot was spread into OF agar and PDA for endophytic fungal isolation. It was carried out in eight replicates in CRD design.
  • These Petri dishes were kept in an incubator at 28 °C for 48 h, and bacterial and fungal counts were carried out using the following equation.
  • Bacterial and fungal counts were calculated using the equation [11]:

CFU mL^-1^ = Number of colonies × dilution factor / volume of suspension spread (0.1 mL)

**3) Production economics of okara-based media and readily available media**

We compared the economics of preparing 1 L of okara-based media with those of readily available NA (Himedia Laboratory, India) and PDA (Himedia Laboratory, India) available in the market. We used the current chemical prices from the Himedia 2025–26 brochure in INR, along with the okara market price of 50 ₹ per Kg (approximately).

## Method validation

Where transparent Petri dishes are PDA and translucent plates are OF agar media.

### Fungal growth

**Fig. 1:**
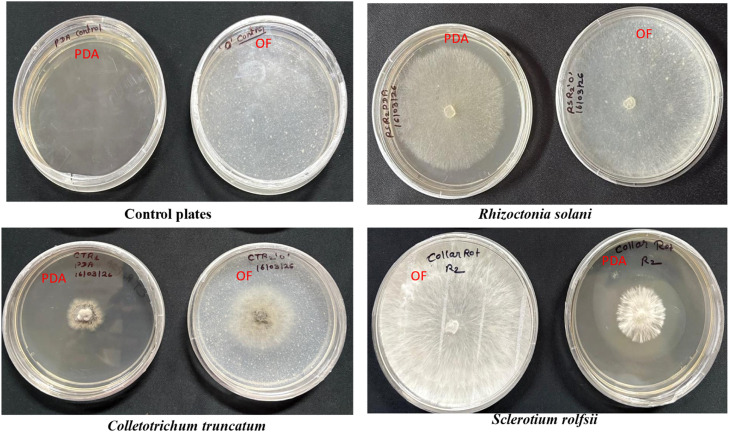
Comparative mycelial growth of fungi on okara fungi (OF) agar and potato dextrose agar (PDA).

**Fig. 2:**
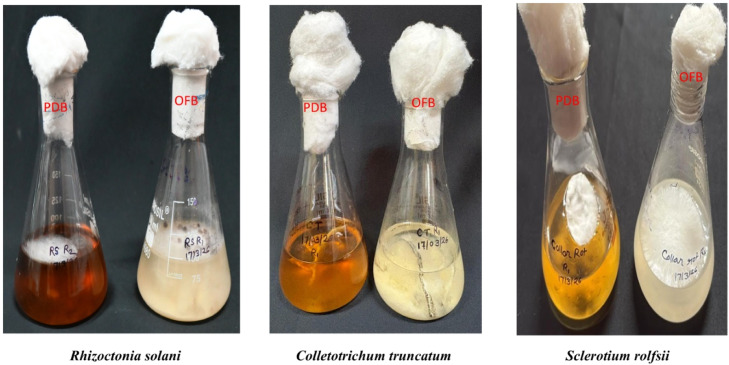
Comparative fungal biomass production in okara fungi broth and potato dextrose broth.

Among all the fungal genera tested, all showed significantly larger fungal diameters grown in OF agar than in PDA (Fig. 1 and Table 1). Similarly, fungal dry weight measurements showed that all these test fungi had significantly higher dry weight when grown in OF broth than in PD broth media (Fig. 2 and Table 2).

**Table 1:** Comparative assessment of fungal colony diameter on okara fungi agar and potato dextrose agar.

Growth media	*Rhizoctonia solani* (cm)	*Sclerotium rolfsii* (cm)	*Colletotrichum truncatum* (cm)
Potato dextrose agar	6.15b	6.10b	4.90b
Okara fungi agar	8.00a	8.00a	7.95a
*p* value (0.05)	< 0.0001	< 0.0001	< 0.001

**Table 2:** Comparison of fungal dry weight in potato dextrose broth and okara fungi broth media.

Growth media	*Rhizoctonia solani* (g)	*Sclerotium rolfsii* (g)	*Colletotrichum truncatum* (g)
Potato dextrose broth	0.451b	0.342b	0.255b
Okara fungi broth	0.871a	0.588a	0.905a
*p-value* (0.05)	< 0.05	< 0.01	< 0.01

Where data are presented as means followed by different letters, indicating significant differences in the treatment analyzed using one-way ANOVA followed by Tukey Honestly Significant Difference (HSD) test at 5 %. Data analyzed through SPSS version 31.

Where fungal diameters are presented as means followed by different letters, they show significant differences in the treatment analyzed using one-way ANOVA followed by Tukey HSD test at 5 %. Data analyzed through SPSS version 31.00.

Where data are presented as means of optical density recorded at 600 nm relative to uninoculated nutrient broth, followed by different letters, indicate significant differences in the treatment analyzed using one-way ANOVA followed by Tukey HSD test at 5 %. Data analyzed through SPSS version 31.00.

### Bacterial growth

**Fig. 3:**
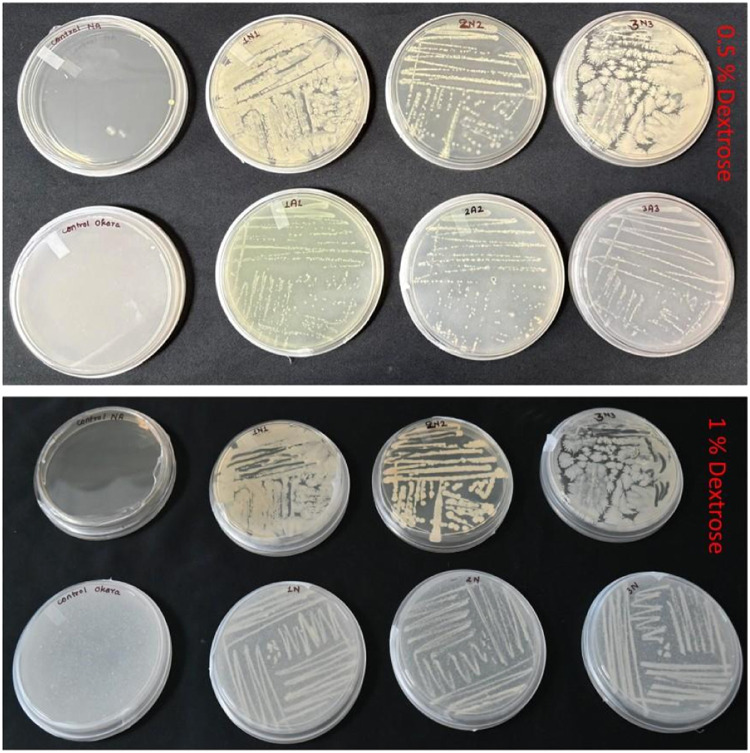
Comparative bacterial growth on Nutrient Agar and Okara bacterial agar at different dextrose concentrations (0.5 % and 1 %).

Three different bacterial genera streaked on NA and OB agar media showed that 1 % dextrose amendment resulted in greater bacterial growth than 0.5 % dextrose, as observed by appearance in Petri dishes. Therefore, we continued with 1 % dextrose amendments for further growth experiment (Fig. 3). Similarly, spectrophotometric absorbance measurements showed that the growth of *Achromobacter* sp. and *Pseudomonas* sp. was similar in both OB broth and NB. However, *B. amyloliquefaciens* growth was significantly lower in the OB broth. It showed that OB could be a cost-effective alternative for NB media (Table 3). However, we can amend it with any critical nutrient so that OB could be a cost-effective alternative for specific bacterial genera or species, as in the case of *B. amyloliquefaciens* in the present investigation.

**Table 3:** Spectrophotometric bacterial growth measurements in nutrient broth and okara bacterial broth media.

Growth media	*Bacillus amyloliquifaciens*	*Achromobacter denitrificans*	*Pseudomonas* sp.
Nutrient broth	0.279a	0.232a	0.818a
Okara bacterial broth	0.164b	0.205a	0.754a
*p-value* (0.05)	0.021	0.405	0.07

Among bacterial and fungal growth in okara-based media, fungi showed greater advantages over bacteria. Therefore, slow-growing fungi such as *C. truncatum* may exhibit significantly enhanced growth when grown in an okara-based medium. It completely grows on Petri dishes in 5 days, whereas PDA took 7 days to complete growing.

### Isolation and enumeration of bacteria and fungi in okara media

Fungal and bacterial counts showed no significant differences between readily available and okara-based media after 48 h (Table 4). We noticed that bacteria appeared earlier on NA than on OB agar during the first 24 h. However, most of the bacteria appeared after 24 h in OB agar. After 48 h, bacterial colonies begin to merge, making counting difficult. In contrast, test fungi growing on OF agar grow more rapidly than those growing on PDA.

**Table 4:** Assessment of bacterial and fungal count in commercially available media versus okara-based (OB) media.

Sample description	Fungal count (× 10^5^) CFU mL^-1^		*p* value (0.05)	Bacterial count (× 10^7^) CFU mL^-1^		*p* value (0.05)
	PDA	OF agar		NA	OB agar	
Soybean rhizosphere soil	1.33a	1.67a	0.52 (NS)	7.00a	6.00a	0.66 (NS)
Soybean root endosphere	1.00a	1.33a	0.37 (NS)	6.00a	4.00a	0.29 (NS)
Wheat rhizosphere soil	3.00a	3.67a	0.58 (NS)	2.67a	1.67a	0.35 (NS)
Wheat root endosphere	1.67a	2.53a	0.42 (NS)	6.00a	4.00a	0.20 (NS)

Where bacterial and fungal counts are presented as means followed by different letters, they show significant differences in the treatment analyzed using one-way ANOVA followed by Tukey HSD test at 5 %. NS indicates a non-significant difference between treatments. Data analyzed through SPSS version 31.00.

### Comparison of the economics of okara-based media and the readily available media

The economics of readily available media and okara-based bacterial or fungal media indicate that okara-based media are a much more cost-effective alternative. Okara-based bacterial and fungal media are 86.27 % and 88.82 % cost-effective, respectively, compared with their respective readily available media (Table 5).

**Table 5:** Production economics of Okara media and readily available media.

constituent	Potato dextrose agar M1941 (price-500 g) ₹	Nutrient agar M001 (price-500 g) ₹	Okara bacterial agar (price-500 g) ₹	Okara fungal agar (price-500 g) ₹
Media	6685	6006	25	25
Sodium chloride	-	-	1615	-
Dextrose	-	-	507	507
Agar-agar	-	-	1174	1174
Per liter media cost	522	469	84	68
**Percent** ₹ **saving**	**-**	**-**	**86.27 %**	**88.82 %**

## Limitations

Since okara is a rich medium, its amendments with dextrose allow the growth of both fungi and bacteria. However, okara-based media are translucent, so anyone who wants to study fungal or bacterial morphology would find a readily available medium preferable. Further, even if initial preheating is followed by straining the okara media through a muslin cloth, some of its constituents settle to the bottom after autoclaving. However, in the future, the above issues may be resolved by adding dispersing agents to prevent coagulation. Our findings showed that a 1 % dextrose amendment supports similar bacterial growth; future amendments of critical growth factors might further enhance bacterial biomass or growth. Okara's nutritive value may vary depending on the soybean variety used for tofu or milk preparation. Therefore, nutritional supplementation with some nutrients might be needed to accelerate microbial growth.

Therefore, despite these limitations, the okara-based media showed better fungal growth and similar bacterial growth to those of the readily available media, suggesting that it can be used for the isolation and enumeration of plant and soil microorganisms. So, mass multiplication of bacteria and fungi for biofertilizer or biocontrol preparation, or for fungal or bacterial sick-plot preparation for screening plant genotypes, can be done at a lower cost. Since okara is rich in nitrogen, adding specific constituents might support growth of other microorganisms like actinomycetes or yeasts. This utilization of okara waste may support research in sustainable microbiology and green lab practices.

## CRediT author statement

Hemant Singh Maheshwari, Laxman Singh Rajput, and Sanjeev Kumar: Conceptualization, methodology, and resources, writing original draft, statistical analysis; Aayushi Singh, Palak Solanki, Rajesh Jewaliya, and Aakash Gour: investigation and recording data; Jeberlin Prabina Bright, Kunwar Harendra Singh & Mahaveer Prasad Sharma: supervision and reviewing the final draft.

## Declaration of competing interest

The authors declare that they have no known competing financial interests or personal relationships that could have appeared to influence the work reported in this paper.

## Data Availability

Data will be made available upon request from the corresponding author.
